# Active surveillance documents rates of clinical care seeking due to respiratory illness

**DOI:** 10.1111/irv.12753

**Published:** 2020-05-16

**Authors:** Marta Galanti, Devon Comito, Chanel Ligon, Benjamin Lane, Nelsa Matienzo, Sadiat Ibrahim, Atinuke Shittu, Eudosie Tagne, Ruthie Birger, Minhaz Ud‐Dean, Ioan Filip, Haruka Morita, Raul Rabadan, Simon Anthony, Greg A. Freyer, Peter Dayan, Bo Shopsin, Jeffrey Shaman

**Affiliations:** ^1^ Department of Environmental Health Sciences Mailman School of Public Health Columbia University New York NY USA; ^2^ Department of Integrative Biology University of California Berkeley CA USA; ^3^ Department of Epidemiology of Microbial Diseases Yale School of Public Health Yale University New Haven CT USA; ^4^ The Earth Institute Columbia University New York NY USA; ^5^ Department of Systems Biology Columbia University New York NY USA; ^6^ Department of Epidemiology Columbia University New York NY USA; ^7^ Department of Pediatrics Columbia University New York NY USA; ^8^ Departments of Medicine and Microbiology New York University School of Medicine New York NY USA

**Keywords:** ILI, medically attended respiratory infections, population‐based estimate of respiratory infections, respiratory illness surveillance

## Abstract

**Background:**

Respiratory viral infections are a leading cause of disease worldwide. However, the overall community prevalence of infections has not been properly assessed, as standard surveillance is typically acquired passively among individuals seeking clinical care.

**Methods:**

We conducted a prospective cohort study in which participants provided daily diaries and weekly nasopharyngeal specimens that were tested for respiratory viruses. These data were used to analyze healthcare seeking behavior, compared with cross‐sectional ED data and NYC surveillance reports, and used to evaluate biases of medically attended ILI as signal for population respiratory disease and infection.

**Results:**

The likelihood of seeking medical attention was virus‐dependent: higher for influenza and metapneumovirus (19%‐20%), lower for coronavirus and RSV (4%), and 71% of individuals with self‐reported ILI did not seek care and half of medically attended symptomatic manifestations did not meet the criteria for ILI. Only 5% of cohort respiratory virus infections and 21% of influenza infections were medically attended and classifiable as ILI. We estimated 1 ILI event per person/year but multiple respiratory infections per year.

**Conclusion:**

Standard, healthcare‐based respiratory surveillance has multiple limitations. Specifically, ILI is an incomplete metric for quantifying respiratory disease, viral respiratory infection, and influenza infection. The prevalence of respiratory viruses, as reported by standard, healthcare‐based surveillance, is skewed toward viruses producing more severe symptoms. Active, longitudinal studies are a helpful supplement to standard surveillance, can improve understanding of the overall circulation and burden of respiratory viruses, and can aid development of more robust measures for controlling the spread of these pathogens.

## 1 INTRODUCTION

Respiratory infections are a leading cause of morbidity and mortality globally and impose a high burden on economic productivity and medical and public health systems (hospitalizations, visits, therapeutics). A variety of viruses and bacteria regularly generate respiratory infections in humans, and specific pharmacological interventions are limited to vaccines, antivirals and antibiotics for a small subset of these pathogens. New and improved vaccines and therapeutics for many common respiratory viruses—respiratory syncytial virus (RSV), rhinovirus, human metapneumovirus (HMPV)—are currently under evaluation or development; however, because many infected persons do not seek clinical care, the true burden of each of these viruses is not known. This circumstance complicates predictive quantification of the cost effectiveness of each intervention and its ability to control targeted pathogens in the broader population.

Household, serological and community studies have shown that a consistent percentage of respiratory infections (most frequently influenza infections) are asymptomatic or subclinical.^1^, ^2^, ^3^ However, estimates of the asymptomatic ratio are highly heterogeneous^3^ and tend to be lower for household studies following a symptomatic index case (10%‐30%) than serologic^1^ and longitudinal community studies,^2^, ^4^ most of which identify the majority of infections as asymptomatic.

Presently, respiratory surveillance in the United States is performed at local scales, is healthcare‐based, and is typically syndromic or viral.^5^ Syndromic surveillance was established mainly to capture influenza activity through data collection on patients seeking care for respiratory symptoms within select facilities. Patient complaints classifiable as influenza‐like illness (ILI, formally defined in the United States as fever plus sore throat and/or cough) are documented regardless of laboratory diagnosis. ILI is a convenient measure and is routinely used to capture seasonal influenza trends in many countries. However, it has been shown that syndromic diagnosis alone cannot establish the etiology of respiratory manifestations because many respiratory viral infections present with similar symptoms.^6^, ^7^ Hence, many influenza surveillance systems and predictive models supplement syndromic surveillance with laboratory‐confirmed diagnosis performed on patient specimens, that is, viral surveillance.^5^, ^8^ However, the number of specimens collected for laboratory assay and reported to public health officials make up a very small subset of the total cases, and many collaborating laboratories test exclusively for influenza.

Syndromic and viral surveillance only draw upon medically attended cases and are neither designed to capture mild or asymptomatic respiratory infections nor to represent the large part of the population that chooses not to seek care. Reports on syndromic and viral surveillance have to be interpreted as ratios (eg, the number of patients with classifiable ILI among total visits within reporting facilities; the number of virus positive specimens among all specimens tested) and thus do not give a broad estimate of prevalence of disease or infections in the general population. In contrast, serology can be performed to assess rates of antibody production in the broader population against a particular virus. However, serological studies are retrospective, and thus unsuitable for estimating prevalence in a timely manner, and indirect, and thus not optimal for viruses eliciting short‐lived immunity.^9^

To estimate the total impact of respiratory illness on the population, multiplicator models based on telephone and web surveys have been used, such as during the 2009 influenza pandemic.^10^, ^11^ Studies have estimated that 17%‐30% of people experiencing ILI seek medical attention during a typical flu season^2^, ^12^, ^13^, ^14^; however, across the world, rates of seeking health care for respiratory symptoms are more heterogeneous and range from 4% to 85%.^15^, ^16^ In New York City (NYC), a survey conducted by the Department of Health and Mental Hygiene (DOHMH) estimated that each Emergency Department‐attended ILI corresponds to roughly 60 illnesses in the (adult) community.^14^ Both survey‐ and web‐based approaches have some important limitations. First, they overlook asymptomatic and mild infections, which are important from an epidemiological vantage. Second, despite being nonspecific, self‐reported ILI is often inappropriately interpreted as an indicator of influenza infection. Third, healthcare‐seeking behavior for respiratory illness is highly variable and is dependent on healthcare policy, socioeconomic background, severity of symptoms possibly related to virus type, and the influence of media and community.

Here, we used a longitudinal study approach to estimate the burden of viral respiratory infections at the population level and to evaluate the typical indicators used by surveillance systems. This analysis is part of a broader study intending to document the prevalence and impact of viral respiratory infections on the NYC population. In a previously published analysis, we showed that more than two‐thirds of respiratory infections are asymptomatic and healthy individuals typically experience multiple infections per year, with children and their caretakers presenting more infections per years than other adults.^15^ Here, on one hand, we were interested in measuring the viral agents captured by ILI (influenza‐like illness) and how well medically attended ILI reflects the burden of viral respiratory infections, of influenza alone, and more generally of respiratory disease in the broader population. On the other hand, by using a very unique dataset, we endeavored to quantify the prevalence of respiratory viral infections and illnesses among the general population and to capture healthcare seeking behavior.

## 2 METHODS

### 2.1 Datasets

We used data from multiple datasets: a longitudinal cohort of NYC residents, a cross‐sectional sample of patients seeking care at three NYC pediatric hospitals, and respiratory surveillance data published by the NYC DOHMH. The longitudinal data were used to (a) quantify the impact of respiratory infection in the population in terms of number of infections, healthcare seeking behavior, and symptoms and (b) to evaluate medically attended cases as indicator of disease burden. Cross‐sectional (pediatric) hospital data were used for comparison with virus prevalence in the longitudinal cohort (children and teenager) population. The DOHMH surveillance data were used for comparison with the syndromic and viral data from the cohort.

#### 2.1.1 Cohort

We enrolled 214 healthy individuals from multiple locations in the Manhattan borough of NYC. Cohort composition is the same as described in Refs. [4, 9] and included children attending two daycares, along with their siblings and parents; teenagers and teachers from a high school; adults working at two emergency departments (a pediatric and an adult hospital); and adults working at a university medical center. The study period spanned two years from October 2016 to April 2018 with some individuals enrolled for a single cold and flu season (October‐April) and others for the entire study period. Participants (or their guardians, if minors) had to provide informed consent after reading a detailed description of the study (CUMC IRB AAAQ4358). Nasopharyngeal swab specimens were collected weekly from each enrolled individual and tested for respiratory viruses. Further, participants completed daily self‐reports rating nine respiratory illness‐related symptoms (fever, chills, muscle pain, watery eyes, runny nose, sneezing, sore throat, cough, chest pain), which were recorded on a Likert scale (0 = none, 1 = mild, 2 = moderate, 3 = severe). The daily report also requested information on whether participants had sought medical attention, stayed home, or taken cold/flu‐related medications (both over‐the‐counter and antibiotics, that are not available in NYC without prescription) as a consequence of their listed symptoms. The longitudinal cohort was obtained using convenience sampling.

#### 2.1.2 Pediatric emergency departments (EDS)

A total of 761 children and teenagers were enrolled at three New York pediatric EDs from August 2016 to June 2018. Patients arriving at one of the pediatric EDs with respiratory complaints (ie, acute illness, asthma) were offered the opportunity to take part in the study and, upon parental consent, tested on site for respiratory viruses.

#### 2.1.3 DOHMH

All clinical laboratories that perform influenza testing on NYC residents and a large sample of NYC laboratory facilities licensed to perform influenza testing report results electronically to the DOHMH. These laboratories provide weekly data on the number of influenza tests requested, positive results by influenza type (when available), as well as data on RSV. Beginning with the 2017‐2018 season, three NYC laboratories also provide the DOHMH test results for other respiratory viruses: adenovirus, coronavirus, HMPV, rhinovirus/enterovirus, and parainfluenza.^8^ All EDs in NYC report the weekly total number of ILI. We used DOHMH ILI and viral data and positivity data during the 2016/2017 and 2017/2018 cold/flu seasons for comparison with the cohort data.

### 2.2 Specimen collection and analysis

Nasopharyngeal samples were collected using minitip flocked swabs. Samples were collected by study coordinators once a week at the physical location of each cohort (irrespective of participant symptoms) and one time only for the ED patients at time of ED visit. Samples were screened with a respiratory viral panel for influenza A (any subtype, A/H1N1, A/H3N2, A/H1N1pdm2009) and B; RSV A and B; parainfluenza (PIV) 1, 2, 3, and 4; HMPV; human rhinovirus (HRV); adenovirus B/E and C; and coronavirus 229E, NL63, OC43, and HKU1.

Sample collection and extraction followed the same protocol as in Refs. [4, 9] and are reported in Text S1.

### 2.3 Statistical analysis

Analysis of longitudinal data was conducted using the total number of positive samples, as well as the number of infection events. We defined an infection (or viral) event as a group of consecutive weekly specimens from a given individual that were positive for the same virus (allowing for a one‐week gap to account for false negatives and temporary low shedding). Medically attended illness (MA), sick days (HOME), and medicine uptake (MEDS) were defined as episodes in which the participants reported seeking care, staying home, or taking medicines for any respiratory symptoms, independent of the etiology. Medically attended ILI (MA ∩ ILI) was defined as episodes in which the participants reported seeking care with symptoms compatible with the US ILI definition. Fever was a self‐reported symptom, and no threshold was specified. Medically attended illnesses, sick days, and medicine uptake associated with a viral event were identified within −3/+7 days from any positive test date during an event in order to account for incubation time. We performed the analysis twice, including and excluding co‐infections, defined as samples testing positive for multiple respiratory viruses.

To determine whether the distribution of viral pathogens differs between the cohort (general) population and healthcare‐based settings, we compared the relative distributions of viruses within the cohort (restricted to children and teenagers) and the pediatric EDs. In doing the comparison, we restricted the samples from the ED to match the time of sample collection from the cohort (October 2016 through April 2018). Moreover, we estimated for each virus v_i_ the conditional probability of seeking medical attention $P\left(\text{MA}|v_{i}\right)$ and we used it to define a scaling factor mapping the prevalence of a specific virus in the medical settings $P\left(v_{i}|\text{MA}\right)$ to the prevalence in the general population $P(v_{i})$ . The scaling was defined using Bayes' theorem:

$$P\left(v_{i}\right)=\frac{P\left(\text{MA}\right)}{P\left(\text{MA}|v_{i}\right)}P\left(v_{i}|\text{MA}\right),$$

where $P\left(\text{MA}\right)$ is the probability of a medically attended respiratory illness (viral or otherwise) and $P\left(\text{MA}|v_{i}\right)$ is the probability of seeking care given infection with virus v_i_. In theory, we can use $P\left(\text{MA}\right)$ and $P\left(\text{MA}|v_{i}\right)$ to map viral infection rates in the broader population to those observed in clinical settings.

Estimates of the seasonal average number of viral respiratory infections per individual were obtained by summing the weekly prevalence of new viral events in the cold/flu season (from October through April of both seasons: May through September were excluded due to limited participation of the cohort in specimen collection during summer months). We used the number of tests performed each week as the denominator, which accounted for missing weekly swabs among cohort participants. The same procedure was used to estimate the average number of ILI events per individual, but, in this case, we incorporated data from the entire year as self‐reporting of symptoms persisted through summer (Figures S2 and S3).

## 3 RESULTS

Complete demographic information is presented in Table S1 of the Supplementary Materials. Depending on the virus, 4%‐20% of infections resulted in an individual seeking medical attention (MA), 7%‐44% were associated with one or more sick days, and 24%‐59% were associated with medicine intake (Table 1 and Table S2) within −3/+7 days of testing positive*.* Similar results were obtained when including co‐infections (Table S3). HRV was associated with the most medical visits, due to its high prevalence, but influenza and HMPV were most likely to result in medical care and sick days (chi‐squared test comparing influenza and HMPV to other respiratory viruses *P* < .01)*.* There were 39 reports of participants taking antibiotics (irrespective of associated RVP results); however, this use of antibiotics was associated with a reported medical consultation in only 22 instances and 16 patients reported taking antibiotics for only 1 or 2 days. Further, participants tested positive for a respiratory virus in 17 of the 39 instances (44%) of reported antibiotic intake.

**TABLE 1:** Outcomes associated with respiratory infections: MA stands for medical attention, HOME for sick days, and MEDS indicates medicine intake for a respiratory illness. P(−|v_i_) indicates the probability of a specific outcome given infection with a particular virus. Estimates are obtained from cohort diaries and samples from October 2016 through April 2018. Viral co‐infections are excluded from the table, and analysis including co‐infections is reported in Table S3

Virus	Episodes	MA	P(MA|v_i_)	95% CI	HOME	P(HOME|v_i_)	95% CI	MEDS	P(MEDS|v_i_)	95% CI
Influenza	27	5	0.19	0.04‐0.33	12	0.44	0.26‐0.63	16	0.59	0.40‐0.78
RSV	27	1	0.04	0‐0.1	4	0.15	0.04‐0.34	9	0.33	0.17‐0.54
PIV	26	2	0.07	0‐0.18	4	0.15	0.02‐0.29	8	0.31	0.13‐0.49
HMPV	20	4	0.20	0.03‐0.38	7	0.35	0.14‐0.56	10	0.50	0.28‐0.72
HRV	243	18	0.07	0.04‐ 0.11	27	0.11	0.07‐0.15	69	0.28	0.23‐0.34
Adenovirus	37	5	0.13	0.04‐0.29	5	0.13	0.04‐0.29	9	0.24	0.11‐0.38
Coronavirus	123	5	0.04	0.01‐0.09	9	0.07	0.03‐0.12	32	0.26	0.18‐0.34

**FIGURE 1:**
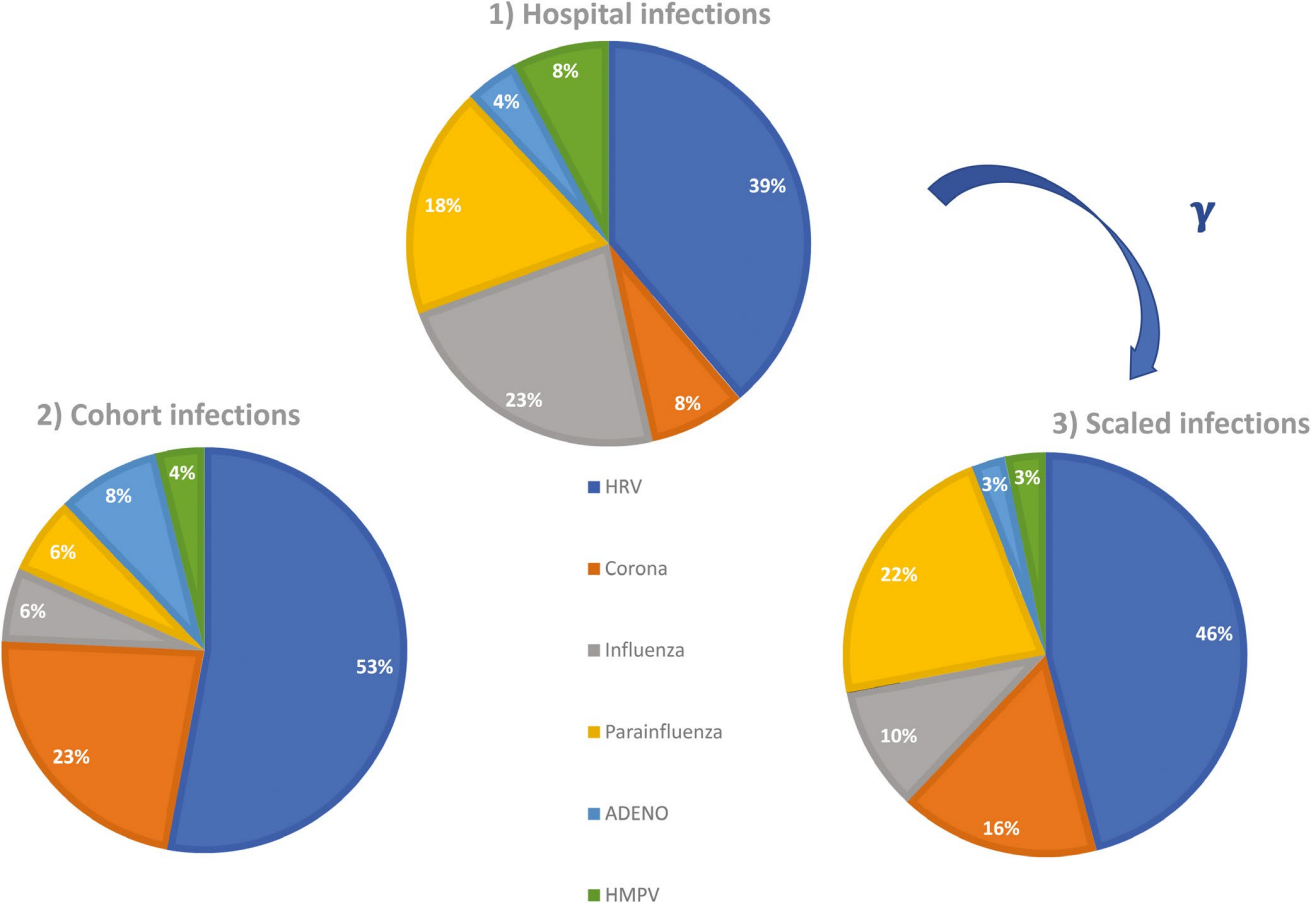
Differences in viral distribution among EDs and the general population. Comparison of the distribution of viruses within patients at pediatric hospitals and among a cohort of children and teenagers tested regularly irrespective of symptoms. The median age associated with specimens was the same for the hospital and cohort (4 y). We restricted the analysis to samples testing positive for a single respiratory virus at PedsED (258) and to samples taken from the children/teenagers cohort (257) within the same time period: October 2016 to April 2018. The pie chart on the right represents data from the pediatric hospitals rescaled by the likelihood of seeking care for a specific virus (Table 1), following the Bayes mapping reported in Methods. We did not consider RSV positivity in either dataset because children in the cohort did not include young infants, who are most subject to severe RSV infections. To estimate the relative proportion of viruses, we can disregard the numerator of the scaling factor and use $P\left(\text{MA}|v_{i}\right)$ from Table 1

The distribution of respiratory viruses differed between the Peds‐ED and the cohort (restricted to children and teenagers) (Figure 1, pie charts 1 and 2), with HRV and coronaviruses making up 76% of total positives in the cohort and 47% in the Peds‐ED. Conversely, influenza and HMPV were, respectively, 23% and 8% of hospital data, but only 6% and 4% of the cohort.

After rescaling the distribution of viruses in the Peds‐ED using Bayes' theorem mapping, that is, the virus‐specific $P\left(\text{MA}|v_{i}\right)$ from Table 1 (see Methods), we obtained more similarly aligned relative distributions of virus types between the cohort and hospital settings (Figure 1 and Figure S1 for analysis including co‐infections). The relative prevalence of type 4 PIV among all PIV differed significantly between the cohort (where PIV4 was the dominant type with 11 counts among 27 PIV events total excluding co‐infections) and hospital data (where PIV4 accounted for only 6 of 48 PIV‐positive test results excluding co‐infections).

Table 2 shows that only a small fraction of the cohort self‐reporting ILI sought medical care: 71% of 159 ILI episodes went unattended. Table 2 also presents the error we would commit in using medically attended ILI (MA‐ILI) as a proxy for viral respiratory infections: Only 2/3 of MA‐ILI were associated with a positive test for (any) respiratory virus within the same week. When restricting to influenza infections, we found that influenza only accounted for 18% of medically attended ILI. Conversely, among cohort participants testing positive for respiratory virus, only 5% of 562 cases both reported seeking medical attention and had symptoms classifiable as ILI. This percentage increases to 36% when conditioning to infections associated with an ILI. Moreover, medically attended ILI did not account for all medically attended respiratory illness: In 54% of 99 medically attended cases, the respiratory symptoms were severe enough to seek care, yet not classifiable as ILI (mostly due to the absence of fever).

**TABLE 2:** Evaluation of MA‐ILI (medically attended ILI) as a measure of respiratory infection, ILI, viral infection, and influenza infection. Estimates are obtained from cohort diaries and samples from October 2016 through April 2018. Symbol ~ denotes negation. A subanalysis is reported in Table S4, where we evaluate the same probabilities using a stricter definition of ILI (typically used by the WHO) requiring specifically fever and cough

Description	Formula	Estimate	95% CI
ILI missed by surveillance	P (~MA|ILI)	0.71	0.64‐0.78
MA‐respiratory disease not recognized by the ILI classification	P (~ILI|MA)	0.54	0.44‐0.64
MA‐ILI without an identified viral infection	P (~infected|MA ∩ ILI)	0.32	0.19‐0.45
MA‐ILI not attributable to influenza	P (~influenza|MA ∩ ILI)	0.82	0.66‐0.92
False negative: viral infection that is not MA‐ILI *if MA‐ILI is a proxy for viral infections	P (~(MA ∩ ILI)|infected)	0.95	0.93‐0.97
False negative: viral infection that is not MA‐ILI *if MA‐ILI is a proxy for influenza	P (~(MA ∩ ILI)|influenza)	0.79	0.62‐0.91
False negative: viral infection accompanied by ILI that is not MA‐ILI *if MA‐ILI is a proxy for viral infections associated with ILI symptoms	P (~(MA ∩ ILI)|i ∩ ILI)	0.64	0.53‐0.75

Each week between 0% and 12% of cohort participants reported symptoms classifiable as ILI (Figure S2), with an average of 2% per week, yielding roughly one ILI per person per calendar year (any etiology). Conversely, an average of 13% of participants tested positive for a new viral infection during weeks in the cold/flu season (Figure S3), indicating between 3 and 4 infections per cold/flu season per person. Table 3 reports the average number of cases of influenza, HRV, coronavirus, HMPV, RSV, PIV, and adenovirus per person per season.

**TABLE 3:** Average number of infections by respiratory virus per person per cold/flu season. Summer months are not included due to decreased participation in the cohort during summer

	HRV	coronavirus	influenza	PIV	adenovirus	HMPV	RSV	All
2016/2017	1.60	1.18	0.21	0.17	0.36	0.08	0.28	3.9
2017/2018	1.76	0.73	0.22	0.09	0.31	0.16	0.14	3.4

Figure S4 shows that the ILI time series for the cohort broadly captures the distribution of official DOHMH reports of ILI‐ED visits in NYC aggregated weekly. Similarly, the timing of the epidemic curves for individual viruses from the cohort data are consistent with those derived from DOHMH surveillance data in NYC and document similarly timed respiratory viral outbreaks (Figure S5 and Text S2).

## 4 DISCUSSION

Medically attended respiratory cases reported to public health systems are only a subset of total respiratory disease. Mild symptoms,^17^ difficulty accessing the healthcare system, lack of perceived risk, and opting for alternative medicine^16^ are typical reasons for not seeking medical care for respiratory symptoms. Estimates of healthcare seeking behavior are typically obtained via telephone or Internet‐based surveys and have been used to develop probabilistic models that transform counts of laboratory confirmed cases to population‐level estimates of prevalence.^10^, ^18^ Overall survey‐estimated rates of physician consultation for respiratory illness vary from 4% to 85%.^15^, ^16^ This considerable variability has been associated with socioeconomic heterogeneity, variable healthcare policies (health insurance policy,^8^ sick leave policy,^15^ health care accessibility), the definition of respiratory illness (typically ILI), variability of pathogen virulence, and the influence of media and social networks during severe epidemics. However, telephone and Internet survey‐based estimates of healthcare seeking behavior are subject to several limitations. First, the surveys are non‐specific: Self‐reported ILI is often used to estimate the burden of influenza. Second, participants in the surveys are typically asked retrospectively to report respiratory illnesses experienced within the last year, a long time‐span for memory recall that can yield misreporting.

Recently, the need for developing methods to link epidemiological studies, clinical practice, and standard surveillance has become a priority.^19^ Here, to contribute to this goal, we used an active, longitudinal sampling study combining daily self‐reported symptoms with weekly laboratory testing for respiratory viruses, and compared these population data to healthcare‐acquired data. The longitudinal data not only allowed estimation of the likelihood of seeking medical attention when experiencing ILI (30%), but also the likelihood of seeking medical attention given infection with individual viruses (a much lower and virus‐dependent probability).

Medically attended ILI has been largely used as a proxy for respiratory infections and most frequently for influenza infections alone. Here, we showed that medically attended ILI is a noisy indicator for the burden of respiratory viruses and for influenza in isolation. In our longitudinal cohort, the majority of ILI events would have gone undetected as most people simply did not seek medical help. Moreover, during weeks in which participants sought care for ILI, 32% tested negative for respiratory viral infection and 82% tested negative for influenza. Among those 32% testing negative, ILI symptoms could have been due to different pathogens (bacteria or viruses not included in the RVP) or to infections manifesting for too few days to be captured by our weekly testing.

Similar estimates for the percentage of ILI due to influenza (found to be 18% here) have been reported both in the literature^20^ and from clinical laboratories reporting to the CDC.^5^ More interestingly, if medically attended ILI is used to estimate respiratory viral infection within the broader population, we find that 95% of infections (and 79% of flu cases) would be unobserved. This result was not only due to the preponderance of asymptomatic infections: Among subjects who tested positive for any virus and developed respiratory symptoms classifiable as ILI, 64% still did not seek medical attention. These observations of limited sensitivity and PPV (positive predicted value) serve as a warning against using ILI in isolation as a proxy index for respiratory virus or influenza prevalence in the population.

Further, the exclusive use of ILI may not be appropriate for capturing medical visits for respiratory illnesses (any cause of disease). In fact, more than half of the medically attended respiratory illnesses reported within the cohort did not satisfy the definition of ILI, mostly because fever was not recorded in cases presenting with other severe symptoms (like cough, chest pain, and sore throat). This misalignment with ILI criteria may be partially due to self‐reporting of symptoms (fever was not necessarily measured) and to the definition of ILI that was specifically designed to capture the signal of influenza. The predictive power of MA‐ILI for influenza was in fact higher than for unspecified respiratory viral infections. We showed in a previous publication^4^ that systemic symptoms (such as fever, chest pain, and muscle pain) are twice more common in influenza and HMPV than the other respiratory viruses. However, a substantial number of severe influenza cases with atypical manifestations or which are simply not classifiable as ILI have been previously documented,^19^, ^21^ indicating an important proportion of influenza disease burden is being overlooked.

The longitudinal sampling approach used here also provides the opportunity to investigate the burden of asymptomatic infections, which standard respiratory surveillance does not document. The preponderance of asymptomatic infections is often overlooked because they do not constitute a direct burden for society; however, these infections likely represent an indirect burden by boosting population‐scale transmission rates (ie, there are additional, asymptomatic contagious persons), helping maintain pathogens within communities, and spawning symptomatic infections (ie, through transmission to individuals who experience more severe outcomes). Further, public health measures such as vaccination, school closure, crowd gathering control, and isolation of infected individuals can only be fully and accurately evaluated following quantification of the total extent of respiratory infection and the role of asymptomatic spreaders.

Data from our cohort showed that within a cold/flu season (October through April) individuals presented with 3‐4 respiratory viral infections on average. (Note, 24% of cohort samples were from children, 9% from teenagers, and the rest from adults below 65 years of age.) Among the many pathogens that cause respiratory symptoms, some (like influenza and HMPV) have been shown to be responsible for more severe infections.^4^ Our observations here support the hypothesis that the distribution of viral prevalence derived from healthcare‐based data is skewed toward more severe pathogens, possibly leading to mis‐estimation of the prevalence of infections in the broader population. The findings, validated through scaling using Bayes' theorem, showed that viruses producing more severe symptoms (influenza, HMPV) were overrepresented in the pediatric ED, whereas others, like coronavirus, were substantially underestimated in prevalence. Further, the substantive difference in prevalence among different types of parainfluenza between hospital and cohort suggests a milder symptom profile for PIV4, which is further corroborated by its reported high rate of seroprevalence in children and young adults despite less frequent isolation.^22^

One of the major limitations of this study is the sample size: Larger longitudinal studies would be required to characterize with greater confidence the likelihood of seeking medical care for individual viruses. Indeed, better estimates could be obtained with a larger sample size by distinguishing among different age‐groups that show different healthcare‐seeking behaviors^23^ and possess different susceptibilities to respiratory infections.^9^ Moreover, as a consequence of the convenience sampling employed here, groups more at risk for serious complications (infants, elders, and immunocompromised) were not represented in our cohort. The fact that some individuals were connected (children attending the same daycare and their family, adults working in the same environment) is another potential bias, as some infections may have been directly transmitted among individuals. Another important limitation is the method used to identify infections: In assessing the potential association between medically attended infections and viral positivity, we considered tests performed within the week, possibly missing some positive results due to the short duration of some respiratory infections. Moreover, we could not account for viruses not included in the viral panel assay.

Clearly, healthcare‐based surveillance is the primary feasible approach for monitoring the prevalence of respiratory viruses regularly and in real time. It also consistently provides critical data supporting infectious disease forecasting efforts, especially for influenza.^24^ However, active longitudinal sampling for respiratory virus infections and care‐seeking behaviors could be used to document important supplementary information largely missed by standard surveillance. This alternate sampling provides a unique picture of respiratory virus prevalence in the community and demonstrates that most respiratory virus infections are not documented as ILI, that rates of seeking clinical care vary by virus, and that many infected individuals seeking care do not meet the definition of ILI. Furthermore, the longitudinal approach can be useful for quantifying potential inappropriate population behavior during respiratory illness (eg, antibiotic uptake without medical consultation).

## DECLARATION OF INTERESTS

JS and Columbia University disclose partial ownership of SK Analytics. JS also discloses consulting for Merck. All other authors declare no competing interests.

## Supporting information

Supplementary Material
