# Supplementary material for: Active surveillance documents rates of clinical care seeking due to respiratory illness
Source: Influenza Other Respir Viruses. 2020 May 16;14(5):499–506. doi: 10.1111/irv.12753 (PMC7276732; doi:10.1111/irv.12753)
Supplement: Supplementary file 1 — Supplementary Material [file IRV-14-499-s001.docx]

**Supplementary Material**

**Table S1: Demographics of the study cohort**

**Text S1: Specimen storage and analysis**

**Table S2: Self-reported medicine intake by virus**

**Table S3: Likelihood of seeking care, staying home and taking medicine when including co-infections with multiple viruses.**

**Figure S1: Differences in viral distribution among EDs and the general population when including coinfections.**

**Table S4: Sub-analysis with WHO alternate ILI definition**

**Figure S2: Self-reported symptoms and ILI in the cohort.**

**Figure S3: Samples and infections in the cohort.**

**Figure S4: Comparison of ILI prevalence within the cohort and official reports from DOHMH.**

**Figure S5: Epidemic curves for HRV and coronavirus during season 2017/18.**

**Text S2: Comparison of ILI population prevalence within the cohort and estimates from DOHMH.**

**References**

**Table S1: Demographics of the study cohort.**

Table is an extension of data published in [1, 2].

|  | Children | | Parents | Teens | Teachers | Peds  ED | Adult ED | Medical Center | All Cohorts |
| --- | --- | --- | --- | --- | --- | --- | --- | --- | --- |
|  | N (%) | | N (%) | N (%) | N (%) | N (%) | N (%) | N (%) | N (%) |
| Enrolled | 35(16.4) | | 20(9.3) | 42(19.6) | 15(7.0) | 22(10.3) | 11(5.2) | 69(32.2) | 214(100) |
| Total samples | 1016(24.1) | | 524(12.4) | 361(8.6) | 248(5.9) | 537(12.7) | 103(2.4) | 1426 (33.8) | 4215(100) |
| Enrollment  (% per cohort)  Year One  Year Two  Both | 8(22.9)  10(28.6)  17(48.5) | | 8(40.0) 6(30.0) 6(30.0) | 33(78.6)  9(21.4)  0(0) | 7(46.6)  1(6.7)  7(46.6) | 11(50.0)  2(9.1)  9(40.9) | 0(0)  11(100)  0(0) | 15(21.7)  41(59.4)  13(18.9) | 82(38.3)  80(37.4)  52(24.3) |
| Samples per individual(median)  Samples per individual(mean) | | 24  29.1 | 23  26.2 | 11  10.7 | 22  20.1 | 20  25 | 9  9.5 | 20  22.6 | 19  21.6 |
| Gender  (% per cohort)  Male  Female  Transgender | 17(48·6)  18(51·4)  0(0) | | 3(15.0)  17(85.0)  0(0) | 27(64.3)  15(35.7)  0(0) | 8(53.3)  7(46.7)  0(0) | 7(31.8)  15(68.2)  0(0) | 4(36.4)  7(63.6)  0(0) | 27(39.1)  41(59.4)  1(1.5) | 93(43.5)  120(56.0)  1(0.5) |
| Age  Range  Median | 0-9  3 | | 24-43  33 | 14-18  14 | 24-38  27 | 24-61  39 | 25-63  33.5 | 20-63  26 | 0-63  25 |
| Hispanic  (% per cohort)  Yes  No  Don’t know | 25(71.4)  10(28.6)  0(0) | | 8(40.0)  10(50.0)  2(10.1) | 20(47.6)  21(50.0)  1(2.4) | 2(13.3)  13(86.7)  0(0) | 1(4.5)  21(95.5)  0(0) | 0(0)  11(100)  0(0) | 12(17.4)  57(82.6)  0(0) | 68(31.8)  143(66.8)  3(1.4) |
| Race  (% per cohort)  White  AfricanAmerican  Asian  AmericanIndian  Other Pacific  Other or mixed  Don’t know | 3(8.6)  3(8.6)  3(8.6)  19(54.2)  0(0)  7(20.0)  0(0) | | 4(20.0)  2(10.0)  3(15.0)  5(25.0)  0(0)  6(30.0)  0(0) | 1(2.4)  21(50.0)  2(4.8)  1(2.4)  1(2.4)  10(23.8)  6(14.3) | 9(60.0)  4(26.7)  1(6.7)  0(0)  0(0)  1(6.7)  0(0) | 18(81.8)  0(0)  3(13.6)  0(0)  0(0)  0(0)  1(4.6) | 5(45.5)  2(18.2)  3(27.3)  0(0)  0(0)  1(9.0)  0(0) | 37(53.6)  4(5.8)  21(30.4)  1(1.5)  0(0)  5(7.2)  1(1.5) | 77(36.0)  36(16.8)  36(16.8)  26(12.1)  1(0.5)  30(14.0)  8(3.7) |

**Text S2: Specimens. Procedure is the same described in** [1, 2]

Samples were stored in 2 ml DNA/RNA Shield (Zymo Research, Irvine, CA) at 4-25°C for up to 30 days and then stored at -80°C in two aliquots. Nucleic acids were extracted from 200 ul of sample and 10 ul internal control using the EasyMAG NucliSENS system (bioMerieux, Durham, NC). Samples were then screened for viruses using the eSensor XT-8 respiratory viral panel (RVP; GenMark Dx, Carlsbad, CA) [3], a multiplex PCR assay. The RVP system separately detects influenza A (any subtype, A/H1N1, A/H3N2, A/H1N1pdm2009) and B; RSV A and B; parainfluenza (PIV) 1, 2, 3, and 4; HMPV; human rhinovirus (HRV); adenovirus B/E and C; and coronavirus 229E, NL63, OC43, and HKU1. Samples positive for a particular virus were identified by an electrical signal intensity of ≥2 nA/mm^2^ (with the exception of coronavirus OC43 for which positive results were identified by an intensity of ≥25 nA/mm^2^, per manufacturer specifications).

**Table S2: Self-reported medicine intake by virus**

Co-infections are excluded from the analysis.

| VIRUS | EPISODES | TYLENOL | IBUPROPHEN | ANTIBIOTIC | COUGH MED | NASAL DECONGEST. | THROAT SOOTHENER | ANTIHISTAMIN | ALTERNATIVE |
| --- | --- | --- | --- | --- | --- | --- | --- | --- | --- |
| Influenza | 27 | 1 | 7 | 5 | 2 | 2 | 1 | 1 | 2 |
| RSV | 27 | 2 | 4 | 1 | 5 | 1 | 3 | 0 | 0 |
| PIV | 26 | 5 | 4 | 1 | 1 | 1 | 2 | 3 | 0 |
| HMPV | 20 | 3 | 5 | 3 | 2 | 5 | 4 | 1 | 3 |
| HRV | 243 | 21 | 35 | 4 | 21 | 15 | 22 | 15 | 7 |
| Adenovirus | 37 | 2 | 6 | 2 | 2 | 1 | 4 | 1 | 0 |
| Coronavirus | 135 | 8 | 15 | 1 | 4 | 12 | 7 | 5 | 1 |

**Table S3. Likelihood of seeking care, staying home and taking medicine when including co-infections with multiple viruses.**

In case of co-infection, the event of seeking care, staying home and taking medicine is attributed to all viruses in the co-infection event.

| VIRUS | EPISODES | MA | P(MA\|v_i_) | 95% C.I. | HOME | P(HOME\|v_i_) | 95% C.I. | MEDS | P(MEDS\|v_i_) | 95%  C.I. |
| --- | --- | --- | --- | --- | --- | --- | --- | --- | --- | --- |
| Influenza | 32 | 7 | 0.22 | 0.08-0.36 | 14 | 0·44 | 0.27-0.61 | 18 | 0.56 | 0.38-0.74 |
| RSV | 30 | 2 | 0.07 | 0-0.16 | 6 | 0·20 | 0.06-0.34 | 12 | 0.40 | 0.22-0.58 |
| PIV | 31 | 3 | 0.10 | 0-0.2 | 4 | 0·13 | 0.01-0.25 | 9 | 0.29 | 0.13-0.45 |
| HMPV | 20 | 4 | 0.20 | 0.03-0.38 | 7 | 0·35 | 0.14-0.56 | 10 | 0.50 | 0.28-0.72 |
| HRV | 268 | 24 | 0.09 | 0.06- 0.12 | 31 | 0.12 | 0.08-0.15 | 70 | 0.26 | 0.21-0.31 |
| Adenovirus | 62 | 9 | 0.14 | 0.06-0.24 | 10 | 0·16 | 0.07-0.25 | 14 | 0.23 | 0.12-0.33 |
| Coronavirus | 135 | 6 | 0.04 | 0.01-0.08 | 13 | 0·10 | 0.05-0.15 | 35 | 0.26 | 0.19-0.33 |

**Figure S1. Differences in viral distribution among EDs and the general population when including coinfections.**

Comparison of the distribution of viruses within patients at pediatric hospitals and among a cohort of children and teenagers tested regularly irrespective of symptoms. We restricted the analysis to samples testing positive for one or multiple respiratory viruses collected at PedsED (323) and events testing positive for one or multiple respiratory viruses collected from the children/teenagers cohort (311) during the same time period: October 2016 to April 2018. The pie chart on the right represents data from the pediatric hospitals rescaled by the likelihood of seeking care for a specific virus (Table 1), following the Bayes mapping reported in Methods.

**Table S4: Sub-analysis with WHO alternate ILI definition.**

Sub-analysis from Table 3 with the alternate definition of ILI (fever + cough) as used in WHO protocols. In the main text we used a definition of ILI consistent with US surveillance (fever +cough and/or sore throat).

| DESCRIPTION | FORMULA | ESTIMATE | 95% C.I. |
| --- | --- | --- | --- |
| ILI missed by surveillance | P (~MA\|ILI) | 0.66 | 0.54-0.64 |
| MA-respiratory disease not recognized by THE ILI classification | P (~ILI\|MA) | 0.61 | 0.50-0.70 |
| MA-ILI withOUT AN identified viral infection | P (~infected \| MA∩ILI) | 0.29 | 0.14-0.48 |
| MA-ILI NOt attributable to influenza | P (~influenza \| MA∩ILI) | 0.77 | 0.59-0.90 |
| FALSE NEGATIVE: viral infection that is not MA-ILI  *if MA-ILI is a proxy for viral infections | P (~(MA∩ILI) \| infected) | 0.96 | 0.94-0.98 |
| FALSE NEGATIVE: viral infection that is not MA-ILI  *if MA-ILI is a proxy for INFLUENZA | P (~ (MA∩ILI) \| influenza) | 0.79 | 0.62-0.91 |
| FALSE NEGATIVE: viral infection accompanied by ILI that is not MA-ILI  *if MA-ILI is a proxy for viral infections associated with ILI symptoms | P (~(MA∩ILI) \| i∩ILI) | 0.58 | 0.43-0.71 |

**Figure S2: Self-reported symptoms and ILI in the cohort**

Time series of ILI within the cohort (solid blue line) represented as a percentage of total weekly reports. The dashed red line indicates the number of reporting participants per week.

**Figure S3 Specimen tested and infections in the cohort**

Time series of infections within the cohort (solid blue line) represented as a percentage of total weekly tests. The dashed red line indicates the number of participants tested per week.

**Figure S4: Comparison of prevalence within the cohort and official reports from DOHMH.**

Blue line represents the fraction of weekly reports that are classified as ILI, orange line shows the total ILI visits in NYC EDs in the same time period. Data on ED-ILI visits are available only for the cold/flu season [4].

**Figure S5 Epidemic curves for HRV and coronavirus during season 2017/18.**

Dashed lines are DOHMH data aggregated weekly, whereas straight lines represent data from the longitudinal study. Official data from DOHMH for viruses other than influenza and RSV are available only for the season 2017-18. We compared weekly time series only for HRV and coronavirus due to the smaller sample sizes for the other viruses. Similarities between community and surveillance-based epidemics have been previously identified in other studies involving self-reported infections [1].

**Text S2 Comparison of ILI population prevalence within the cohort and estimates from DOHMH.**

We compared the DOHMH estimate of NYC total community ILI (each visit to the emergency department is mapped onto 60 community ILI) [5] to the cumulative ILI-rate from the cohort rescaled to the NYC population. There was good agreement (5 million vs 4·6 million) for season 2017-18 and a relative difference of 1/3 for 2016/17 (5·1 vs 3·3 million). Our estimate of 0·5 ILI per cold/flu season per person was stable across the two seasons whereas hospitalization rates were 1·5 times higher in 2017/18. The higher severity of the 2017-18 flu season could be partially responsible for the divergence. Our cohort is not representative of all risk groups, and media coverage during the more severe 2017/18 flu outbreak may have influenced the ratio of ED versus community ILI. Healthcare seeking behavior has been shown to be deeply influenced by media coverage of severe epidemics, e.g. an estimated 32% to 58% of ILI were medically attended during the 2009 pandemic [3,4], roughly two times the rate for a typical flu season.

**References**

| [1] | Galanti M, Birger R, Ud-Dean M et al., Rates of asymptomatic respiratory virus infection across age groups, *Epidemiology and infection,* 2019; 147:e176, |
| --- | --- |
| [2] | Galanti M, Birger R, Ud-Dean M et al. Longitudinal active sampling for respiratory viral infections across age groups. *Influenza Other Resp Viruses,2019;* 13(3):226-232. |
| [3] | Popowitch EB, O'Neill SS, Miller, MB. Comparison of the Biofire FilmArray RP, Genmark eSensor RVP, Luminex xTAG RVPv1, and Luminex xTAG RVP Fast Multiplex Assays for Detection of Respiratory Viruses. *Journal of Clinical Microbiology*, 2013; 51:1528-1533. |
| [4] | DOHMH, "https://www.health.ny.gov/diseases/communicable/influenza/surveillance/2017-2018/flu_report_current_week.pdf. 2018," New York, 2018. |
| [5] | Metzger KB, Hajat A, Crawford M, Mostashari F. How many illnesses does one emergency department visit represent? Using a population-based telephone survey to estimate the syndromic multiplier. *MMWR Morb Mortal Wkly Rep*, 2004*:* 53:106-111. |
| [6] | Brooks-Pollock E, Tilston N, Edmunds WJ et al. Using an online survey of healthcare-seeking behaviour to estimate the magnitude and severity of the 2009 H1N1v influenza epidemic in England. *BMC Infect Dis*, 2011; 11:68. |
| [7] | Reed C, J Angulo F, Swerdlow D et al. Estimates of the Prevalence of Pandemic (H1N1) 2009, *Emerging infectious diseases.* 2009;15: 2004-7. |
| [8] | Bexelius C, Merk H, Sandin S et al. Interactive Voice Response and web-based questionnaires for population-based infectious disease reporting, *European journal of epidemiology,* 2010; 25:693-702. |
